# Supplementary material for: Optical Control of Young’s Type Double-slit Interferometer for Laser-induced Electron Emission from a Nano-tip
Source: Sci Rep. 2017 Oct 4;7:12661. doi: 10.1038/s41598-017-12832-3 (PMC5627254; doi:10.1038/s41598-017-12832-3)
Supplement: Supplementary file 1 — Supplemental Information [file 41598_2017_12832_MOESM1_ESM.pdf]

Supplemental Information for ”Optical Control of Young’s Type Double-slit Interferometer for Laser-induced Electron Emission from a Nano-tip”

Hirofumi Yanagisawa[1,2,3], Marcelo Ciappina[4], Christian Hafner[5], Johannes Schötz[2,3], Jürg Osterwalder[6], Matthias F. Kling[2,3]

[1] *Institute for Quantum Electronics, ETH Zürich, CH-8093 Zürich, Switzerland*

[2] *Max Planck Institute of Quantum Optics, D-85748 Garching, Germany*

[3] *Physics Department, Ludwig-Maximilians-Universität Munich, D-85748 Garching, Germany*

[4] *Institute of Physics of the ASCR, ELI-Beamlines, Na Slovance 2, 182 21 Prague, Czech Republic*

[5] *Laboratory for Electromagnetic Fields and Microwave Electronics, CH-8092 Zürich, Switzerland*

[6] *Physik-Institut, Universität Zürich, CH-8057 Zürich, Switzerland*

1. Intensity profile of far-field wave function for initial wave packets with short transverse extent.

2. Intensity profile of far-field wave function for initial wave packets with broad temporal width.

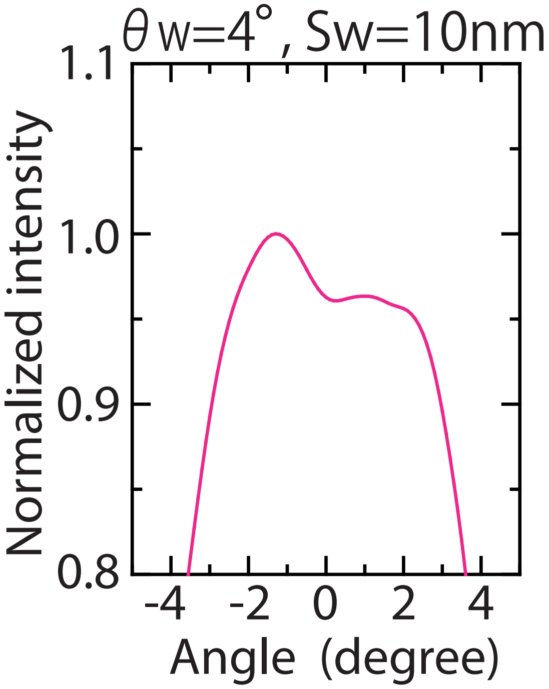

Figure 1: **Figure S1: Far-field intensity distributions.** Simulated far-field intensity distributions. The dimensions of the initial wave functions are $\theta_w = 4^\circ$ and $S_w = 10\,\mathrm{nm}$. The energy of initial wavefunction is 4 eV.

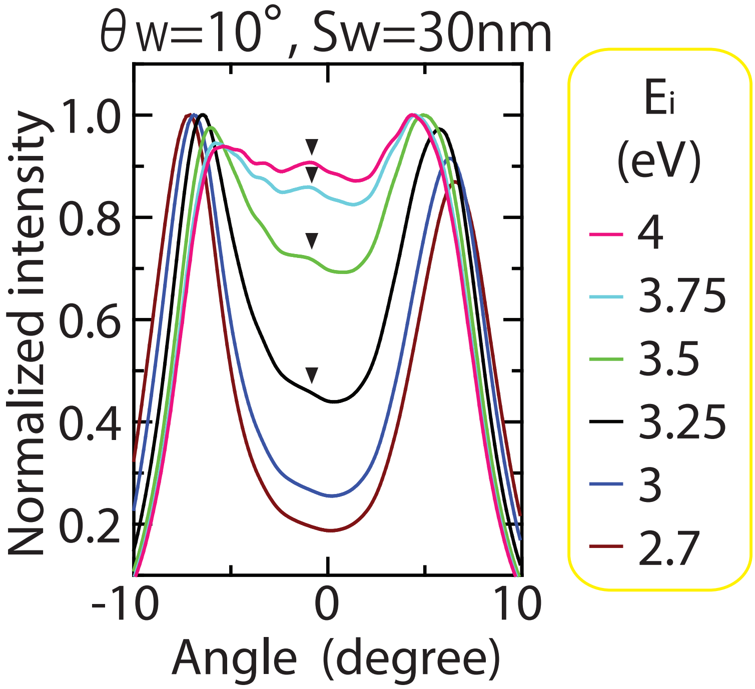

Figure 2: **Figure S2: Far-field intensity distributions for different initial energies.** Simulated far-field intensity distributions as a function of angle with respect to the center axis for various initial energies, which are indicated in the inset. The dimensions of the initial wave functions are $\theta_w = 10^\circ$ and $S_w = 30\,\mathrm{nm}$.
